# To read or not to read – A cross-sectional study of Swedish primary care patients’ adoption of patient accessible electronic health records

**DOI:** 10.1177/20552076241287636

**Published:** 2024-10-07

**Authors:** Irene Muli, Åsa Cajander, Helena Hvitfeldt, Ylva Trolle Lagerros, Daniel Söderberg, Linnea Sjöblom, Anna Dahlgren, Bo C. Bertilson, Nasim Farrokhnia, Isis Amer-Wåhlin, Marina Taloyan, Maria Hägglund

**Affiliations:** 1 Participatory eHealth and Health Data Research Group, Department of Women's and Children's Health, 8097 Uppsala University, Uppsala, Sweden; 2 195561 Department of Information Technology, 8097 Uppsala University, Uppsala, Sweden; 3 72237 Norrtälje Hospital, Vårdbolaget Tiohundra, Stockholm, Sweden; 4 Division of Clinical Epidemiology, 271345 Department of Medicine Solna, 27106 Karolinska Institutet, Stockholm, Sweden; 5 Center for Obesity, Academic Specialist Center, 7674 Stockholm HealthCare Services, Stockholm, Sweden; 6 Division of Family Medicine and Primary Care, 163074 Department of Neurobiology, Care Sciences and Society, 27106 Karolinska Institutet, Solna, Sweden; 7 Academic Primary Healthcare Center, 7674 Stockholm Healthcare Services, Stockholm, Sweden; 8 411435 Department of Clinical Science and Education, Södersjukhuset, 27106 Karolinska Institutet, Stockholm, Sweden; 9 Medical Management Centre, Department of Learning, 411412 Informatics, Management and Ethics, 27106 Karolinska Institutet, Stockholm, Sweden; 10 59561 Uppsala University Hospital, Uppsala, Sweden

**Keywords:** Patient accessible electronic health records, adoption, primary care, survey

## Abstract

**Objective:**

Patient-accessible electronic health records (PAEHR) were implemented in the Stockholm region of Sweden seven years ago. This study examines socio-demographic and psychographic factors associated with reading/not reading these records, as well as the common reasons for such behaviours.

**Methods:**

Patients or guardians of minors seeking face-to-face or digital primary healthcare in the Stockholm region responded to a questionnaire about whether they were aware that they could read their PAEHR, and if so, if they had read it and reasons for reading or not reading. We conducted a comparative analysis of readers and non-readers and a stepwise multiple logistic regression.

**Results:**

The majority of participants were aware that they could read the PAEHR (86%) and among those aware, 77% had read it. The odds of reading decreased with increased age, unfavourable opinion of PAEHR, low information literacy and being single. Access to a smartphone increased the probability of reading. Participants who had read their PAEHR had commonly read it to get an overview of their health and care (65%) and to follow up on a healthcare visit (55%). Participants who had not read their PAEHR stated generally that they did not need to (63%) and/or had received sufficient information from their providers (38%).

**Conclusions:**

While most people were aware they could read the PAEHR and many had read it, a digital divide and several barriers to reading the PAEHR were identified. Efforts to increase PAEHR reading may be targeted at older people, people needing more informal support, those who may be excluded due to limited information literacy, and towards improving the patient portals’ usability.

## Introduction

Increasingly, healthcare systems are shifting towards more digital healthcare but there are concerns about the digital divide where certain groups of people may be excluded from these advancements.^1^ One of the digital healthcare services gradually being implemented globally is patient-accessible electronic health records (PAEHR).^2–5^ PAEHR gives patients online access to their electronic health records (EHR). In Sweden, PAEHR has been available through the national patient portal 1177.se since 2016.^6^ To access the service, authorization is required using a personal electronic identification. Access is granted to patients aged 16 and above, with the possibility for younger patients to apply for access from age 13.^7^ Parents have access to their children's records from birth to age 13. The information available to patients may differ depending on which region they have received care in. For example, not all regions in Sweden give patients online access to EHRs from psychiatric clinics.^8^

Online record access (ORA), through a PAEHR, offers several advantages, including reduced anxiety, better patient–provider relationships and improved medication adherence.^9^ Minorities and older patients have reported more benefits from reading their EHRs to prepare for a healthcare visit than other users.^10^ According to a national survey of users of the Swedish PAEHR, the main reasons for reading were to get an overview, to follow up on health care visits and to become more involved in one's health care, while the least common reason was suspected inaccuracies.^11^

Despite the reported benefits of ORA, few studies have investigated the adoption of PAEHR in the general public. PEHR adoption is here defined as patients using the PAEHR, which can include reading the records. Adoption and use of PAEHRs is often measured by the number of times a user logs in to the PAEHR.^12^ Logging in to the PAEHR does not mean patients are reading their records, which is a specific type of portal use. In this study, we will focus on patients’ reading the PAEHR specifically, and therefore use the terms readers and non-readers to signify the studied patient groups.

Awareness and adoption are also two distinct concepts, and awareness does not necessarily lead to PAEHR use. According to one study investigating whether digital health literacy predicted awareness and use of the Australian PAEHR in the general population, the majority (64%) were aware of the service but only 6% were users and 10% intended to use it.^13^ Other studies of use focused on specific healthcare systems, use within a 12-month period or use in a specific disease group.^10,14,15^

A comprehensive systematic review investigating factors influencing the use of PAEHR among patients found that internet access and use, education, perceived usefulness and income positively influenced subjective- and objective-measured use.^16^ Digital health literacy and health literacy have also been associated with use and intention to use.^13^ The more general concept of information literacy can be defined ‘as an understanding and set of abilities enabling individuals to recognize when information is needed and have the capacity to locate, evaluate and use effectively the needed information’.^17^ Whereas health literacy is important when studying patients understanding of their health records,^17,18^ general information literacy may be equally important for patients’ adoption and use of PAEHRs. Privacy and security concerns have been negatively associated with PAEHR use and intention to use.^16^ Further, according to the Unified Theory of Acceptance and Use of Technology (UTAUT), four main factors influence individual intention and use of technology; Performance Expectancy, Effort Expectancy, Social Influence; and Facilitating Conditions.^19^ Unified Theory of Acceptance and Use of Technology also identifies gender, age, experience and voluntariness, habits, hedonic motivation and price value as influential factors.^20^ More recent studies investigating factors influencing use based on UTAUT found all the main factors influenced intention to use PAEHR in different ways.^21,22^

While several studies have investigated the non-use of platforms providing PAEHR, few have investigated the reasons for non-use of the PAEHR itself. According to one of the few studies investigating reasons for not reading PAEHR in an American context, the main reasons were forgetting to read, or being unaware that the PAEHR was available.^10^ Perceiving it to be unnecessary for health, having received all the information needed from their healthcare provider, and preferring oral information to record access, have also been identified as reasons for non-use.^23^

Despite the existing body of research, no studies have been found investigating adoption of PAEHR in the Swedish population, despite Sweden having a well-established national patient portal and a mature PAEHR service. Similarly, no studies have been found comparing the socio-demographic differences of readers and non-readers of PAEHR among Swedish patients and their reasons for not reading their records online. Previous survey studies^3,11^ exclusively investigated readers’ experiences, failing to increase our understanding of non-readers and how they differ from readers. Thus, there is a research gap regarding awareness and reading, socio-demographic factors associated with reading or not reading health records online, as well as the common reasons for such behaviours, particularly related to the Swedish PAEHR.

Based on the identified research gap, this study explores the factors associated with the adoption of PAEHRs among primary care patients in Sweden. This involves examining to what extent patients actually read the PAEHR, identifying factors associated with reading or not reading these records and understanding the common reasons for such behaviours. Furthermore, identifying the reasons for reading and not reading the PAEHR can enhance our understanding of barriers to adoption and highlight areas for improvement to increase PAEHR usage. Specifically, this study addresses the following research questions:

1. What percentage of primary care patients in our study sample have read or not read their EHR online? (Q1)
2. What are the socio-demographic and psychographic differences between readers and non-readers in terms of age, education, marital status, self-reported health, internet use and access to information and communications technology (ICT) devices, as well as their opinions of PAEHR and self-reported information literacy? (Q2)
3. Which of selected factors are confounders, are significantly associated with and can explain PAEHR reading? (Q3)
4. What are the common reasons for the reading or not reading PAEHR among patients, focusing on perceived utility, accessibility and personal preference? (Q4)

## Methods

We used a cross-sectional study design, and the data was collected through a survey. The survey was developed in discussion with researchers with different expertise. Most items were adapted from a British survey and a previous Swedish survey on PAEHR.^11,24^ The researchers formulated items measuring age, awareness and reading of PAEHR. Items measuring education, marital status, self-reported health, internet use and accessible ICT devices came from two other Swedish surveys.^25,26^ Items investigating self-reported information literacy and common reasons for reading were adapted from the previous Swedish survey on PAEHR.^11^ The section on information literacy was shortened significantly to ensure the questionnaire length would not negatively affect response rates. Four questions related to the study participants’ ability to find, select, understand and judge the reliability of information was included to capture information literacy. Items measuring common reasons for not reading were designed based on a previous study on PAEHR use.^27^ The survey was in Swedish and was validated with seven individuals using cognitive interviewing, leading to minor changes. The survey was available in both paper and online formats.

We reference the UTAUT to discuss our findings within a broader theoretical context. However, UTAUT was not used as a guiding framework in the actual analysis of our empirical data. We used UTAUT to discuss our understanding of the factors influencing the adoption of PAEHR, such as Performance Expectancy, Effort Expectancy, Social Influence and Facilitating Conditions, but the empirical analysis focused on data-driven insights.

### Study sample

The study sample consisted of both patients (18+ years old) and guardians of minors who either had a face-to-face consultation at one of six participating healthcare centres in the region Stockholm or who had a digital consultation through either the public online healthcare application (Alltid öppet) or one of three participating tax-funded, but privately managed online healthcare applications (Doktor24, Kry, Min Doktor). The six participating healthcare centres served communities with low to higher socio-economic status. Initially, all types of consultations who were not related to COVID-19 were included, but a limitation to consultations with physicians was made in the early stages of data collection, since most consultations through the private online care applications were with a physician. Patients whose visits were due to COVID-19 were excluded in order to minimize the impact of the pandemic on the results.

### Data collection

The data was collected between October 2020 and May 2021. At all but one healthcare centre, study personnel approached patients and guardians of minors with information about the study in the waiting room. At one of the healthcare centres, at their request due to concerns for COVID-19 transmission, the reception staff invited and provided interested people with the paper survey. Patients and guardians of minors recruited from the healthcare centres were invited to either fill out a paper survey on-site or take it home and send it back using a prepaid envelope, or provide an email address to receive a link to a digital survey. The online care providers recruited participants receiving digital care through a message on the application or an email with a link to the survey, with an option to request a paper survey sent home. The participants were asked for their email addresses to facilitate reminders. All participants who provided an email address were sent several reminders. Participants were provided with information about the study and were asked to check a box at the end of the page to indicate their consent to participate. While most checked the box and provided consent, some did not. However, since those who did not check the consent box had completed and submitted the survey, we interpreted this as they wished to participate but simply overlooked the consent box. We sought approval to use the surveys with unanswered consent requests, and a waiver was granted by the Swedish Ethical Review Authority (reference number 2021-04602).

Of all paper surveys distributed at the healthcare centres, 38% were filled in and returned. Among participants from the healthcare centres provided with a link to the digital survey, 56% responded. In comparison, of participants who clicked on the link to the digital survey among participants seeking digital care, 41% responded. The total number of people invited to participate was not registered. In total, 66% of the participants participated through the digital survey and 33% through the paper survey. Among readers, as described below, 70% participated through the digital survey and 30% through the paper survey while 57% of the non-reader participated through the digital survey and 43% through the paper survey. For this study, only participants living in Region Stockholm who had a consultation with a physician were included in this analysis (*n* = 3421). A total of 414 participants lived in other regions, and 559 had consultations with a healthcare professional (HCP) other than a physician and were thus excluded from this analysis.

### Statistical analysis

After data collection, before the analysis, data cleaning was conducted by a statistical consultant, and only relevant variables were extracted and provided from the main dataset for the analysis of this study. Research questions 1, 2 and 4 are descriptive and comparative presented by percentages and prevalence. Differences between the PAEHR readers and non-readers in socio-demographic and psychographic factors/variables are analysed by Pearson chi-square significant test and *t*-test. Research question 3, association between the PAEHR reading and other factors/variables are calculated by stepwise forward multiple logistic regression and presented as odds ratios with 95% confidence intervals.

#### Patient-accessible EHR reading

The survey included questions to determine whether participants were aware of their ability to access their EHRs online and whether they had actually read them. To quantify PAEHR awareness, we asked the respondent if they were aware they could read their EHR online with the response option ‘yes’ or ‘no’ (Appendix A). Although awareness is not the main focus of this study, we report on the proportion of study participants who were not aware they could read their records as this is relevant to understand the overall adoption of PAEHRs in Sweden. To investigate how many of them had read their records, we asked whether they had ever read the PAEHR. Participants responding ‘yes’ (readers) and participants responding ‘no’ (non-readers) were thereafter compared to each other. Only answers from participants responding that they were aware of PAEHR were used in the comparative analysis. To maintain our focus on patients’ reading of PAEHRs, factors influencing patients’ awareness were not further analysed in this study, but may be explored in future work.

#### Socio-demographic and psychographic factors associated with PAEHR reading

Readers and non-readers were compared regarding gender, age, education, marital status, self-reported health, internet use and accessible devices (ICT the participants had access to) (Appendix A). Study participants’ general opinion of having ORA through the PAEHR and self-reported information literacy, that is, the ability to retrieve information, select, understand and judge the reliability of information was also compared. Participants responding ‘other/don’t know/don’t want to answer’ categories regarding gender, education and marital studies were excluded. Participants’ age reflected their age in 2020, education reflected their highest attained education (primary education vs. high school vs. university or college) and self-reported health reflected their general health (Good/very good vs. neither/nor vs. bad/very bad). Marital status measured current status (married, registered partner or stable partner vs. single). Accessible device measured which devices study participants had access to (smartphone vs. phone without apps, computer, tablet or none) since a previous study indicated that the Swedish PAEHR was mainly accessed through a mobile phone^12^ and since the vast majority of the participants (97%) had a smartphone. Internet use measured how often internet was used on different devices (daily vs. Every week or Every month, Less frequently or Never). Opinion of PAEHR reflected whether participant thought it was good for them to read their health records (Strongly agree/agree vs. partially agree/disagree). Self-reported information literacy reflected self-rated abilities (high vs. low). All but one variable (age) was categorical.

Potentials for significant differences in comparative analyses were tested using chi-square and t-test depending on variables. Significant differences that were identified were further explored using stepwise forward multiple logistic regression using a likelihood ratio statistic, to control for confounding factors. Regressions were run automatically in the statistical program Stata which performs a forward selection of the selectable independent variable using the likelihood ratio statistic. The dependent variable for the PAEHR reading (Yes = 1, No = 0) and the selectable independent variables were age, education, marital status, internet use and access to ICT devices, as well as their opinions of PAEHR and self-reported information literacy items. Significance was considered at *p* < 0.05. All statistical analyses were conducted using Stata version 17.0 (Stata Corporation, College Station, TX, USA).

#### Common reasons for reading and not reading the PAEHR

To investigate common reasons for reading, participants who reported reading the PAEHR were asked about their main reasons for reading. Similarly, participants who were aware of PAEHR, but who reported never reading it, were asked about their main reasons for not reading. Both questions had multiple options, including a free-text option ‘other’. Participants could select up to five reasons for reading or not reading. The reasons were ranked based on the percentage of participants that had selected them of all participants that answered the question. The free-text options were coded qualitatively and only the most common responses were presented.

Before the investigation, this study received ethical approval from the Swedish Ethical Review Authority (reference number 2020-00860, 2023-02516-01 and amendments 2020-06506, 2021-04602).

## Results

### Percentage of primary care patients in our study sample have read or not read their EHR online (Q1)

The investigation of PAEHR reading found that most of the participants 86% (*n* = 2751/3208) were aware that they could read their EHR online and 77% (*n* = 2086) of them had read their records (readers), while 23% (*n* = 632) had not read them (non-readers). These findings indicate a high level of awareness among the population studied, which is important for understanding subsequent usage patterns. To maintain our focus on patients’ PAEHR reading, factors influencing patients’ awareness were not further analysed in this study, but may be explored in future work.

### Socio-demographic and psychographic differences between readers and non-readers (Q2)

The comparison of readers and non-readers showed a larger proportion of females among readers than among non-readers (Table 1). Readers of PAEHR were on average younger than non-readers with a mean age of 48 years and had a larger proportion of participants with university or college education and partnered participants compared to non-readers. While almost all participants had access to a smartphone and used internet every day, readers had a larger proportion of people with smartphone access and who use the internet daily compared to non-readers. Additionally, a larger proportion of readers reported it was ‘good for them to read their record’ compared to non-readers. Readers were also more likely to report high self-rated information literacy, that is, ability to retrieve health information from different sources, select needed information, understand, share and judge information reliability. The difference regarding self-reported health between readers and non-readers was not significant (Appendix B) and is thus not illustrated in the figures.

**Table 1.** A comparison of readers and non-readers regarding socio-demographic and psychographic factors.

	Total	Has read, *n* = 2086 (77%)	Has never read, *n* = 632 (23%)	p*
Gender				<0.001
Female	1872 (70%)	1473 (71%)	399 (64%)	
Male	821 (30%)	594 (29%)	227 (36%)	
Mean Age	50	48	56	<0.001
[Min-Max]	[17–96]	[17–90]	[18–96]	
Education				0.014
Primary education	192 (7%)	131 (6%)	61 (10%)	
High school	842 (31%)	648 (31%)	194 (31%)	
University or college	1660 (62%)	1289 (62%)	371 (59%)	
Marital status				0.033
Married, registered partner or Stable partner	2009 (75%)	1559 (77%)	450 (72%)	
Single	654 (25%)	481 (23%)	173 (28%)	
Health				0.561
Good or very good	1943 (72%)	1487 (71%)	456 (72%)	
Neither good nor bad	542 (20%)	414 (20%)	128 (20%)	
Bad or very bad	226 (8%)	180 (9%)	46 (7%)	
Internet use				<0.001
Daily	2674 (98%)	2074 (99%)	600 (95%)	
Every week or Every month, Sometimes or Never	44 (2%)	12 (1%)	32 (5%)	
Accessible devices				<0.001
Smartphones	2640 (97%)	2055 (99%)	585 (93%)	
Just phone without apps, computer, tablet or non	76 (3%)	29 (1%)	47 (7%)	
It is good for me to read				<0.001
Strongly agree/agree	2036 (93%)	1938 (94%)	98 (72%)	
Partially agree, disagree/strongly disagree	161 (7%)	122 (6%)	39 (28%)	
The ability to retrieve health information				<0.001
High	1813 (67%)	1442 (69%)	371 (59%)	
Low	894 (33%)	638 (31%)	256 (41%)	
The ability to select information				<0.001
High	1765 (65%)	1414 (68%)	351 (56%)	
Low	937 (35%)	662 (32%)	275 (44%)	
The ability to understand information				<0.001
High	1896 (70%)	1498 (72%)	398 (63%)	
Low	804 (30%)	575 (28%)	229 (37%)	
The ability to judge the reliability of information				<0.001
High	1823 (67%)	1440 (69%)	383 (61%)	
Low	881 (33%)	638 (31%)	243 (39%)	

*The *p*-value was determined using chi-square and *t*-test depending on the variable.

### Factors that are confounders, are significantly associated with and can explain PAEHR reading (Q3)

The stepwise logistic regression conducted to control for confounding factors indicated that age, opinion of PAEHR, ability to select information, marital status and access to a smartphone significantly predicted PAEHR reading (Table 2). The odds of reading decreased with increased age (0.97, 95% CI 0.95–0.98), negative opinion of PAEHR (0.24, 95% CI 0.15–0.39), low information literacy (0.54, 95% CI 0.37–0.79) and for single people (0.66, 95% CI 0.44–0.99), while the odds for reading increased for people with access to a smartphone (3.39, 95% CI 1.55–7.40).

**Table 2.** Summary of the stepwise logistic regression predicting the odds ratio, with 95% confidence intervals, for reading PAEHR.

	Odds ratio [95% confidence interval]	*p* value
Age	0.97 [0.95–0.98]	<0.001
It is good for me to read		<0.001
Strongly agree/agree (ref)		
Partially agree/disagree	0.24 [0.15–0.39]	
Ability to select info		
High (ref)		0.002
Low	0.54 [0.37–0.79]	
Marital status		0.043
Married, registered partner or Stable partner (ref)		
Single	0.66 [0.44–0.99]	
Accessible device		0.002
Just phone without apps, computer, tablet or non (ref)		
Smartphone	3.39 [1.55–7.40]	

### Reasons for the reading or not reading PAEHR (Q4)

**Figure 1.**
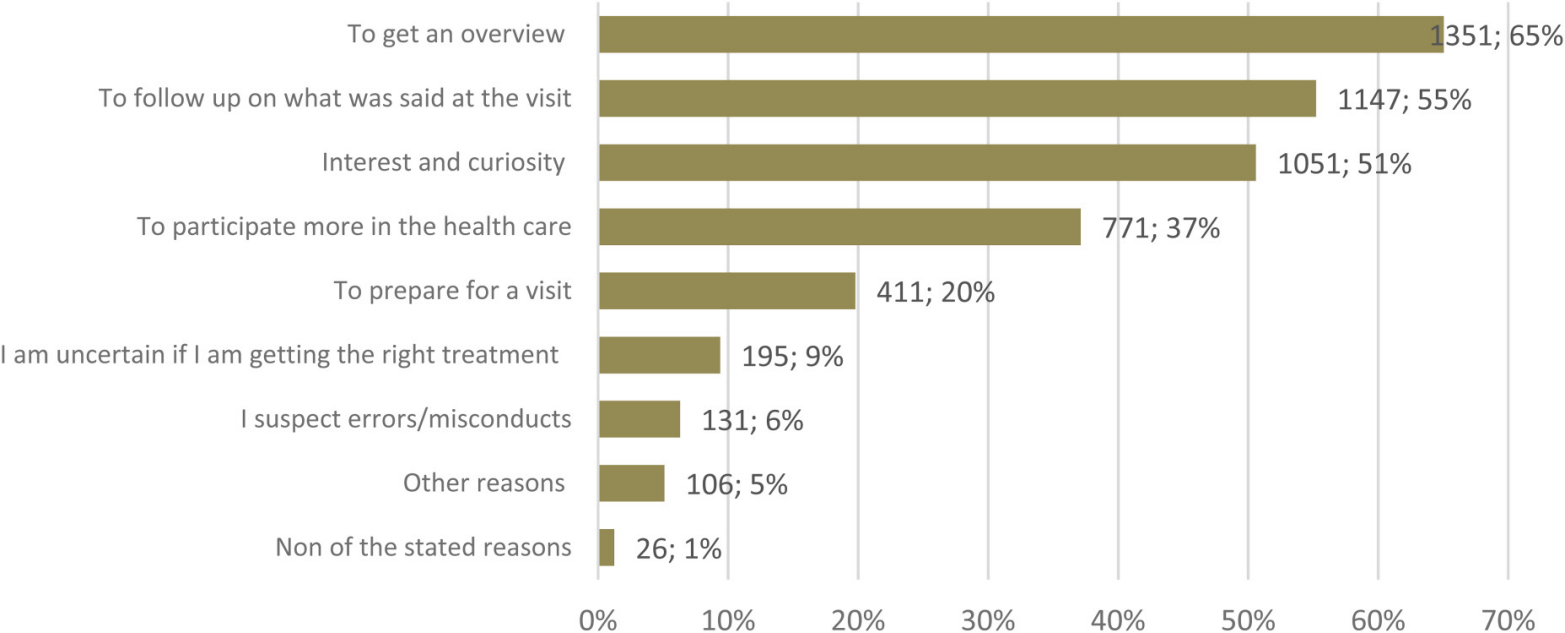
Reasons for reading PAEHR among readers (*n* = 2077).

The investigation of common reasons for reading or non-reading determined that the two reasons indicated by majority of the readers were to get an overview of health and healthcare (65%, *n* = 1351), and to follow up on healthcare visits (55%, *n* = 1147) (Figure 1). Some of the participants (5%, *n* = 106) had provided other reasons, whereof the most common response was to view test results.

**Figure 2.**
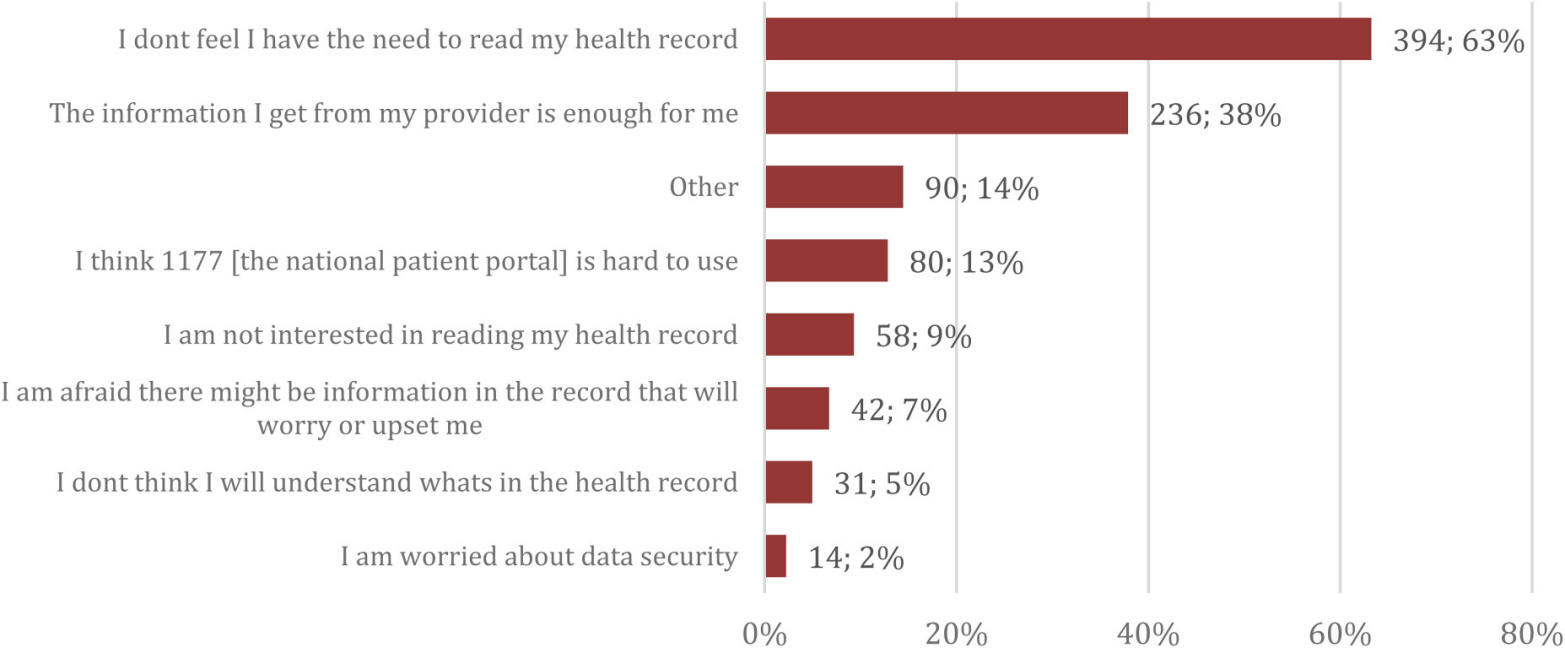
Reasons for not reading PAEHR among the non-readers (*n* = 623).

The primary reason for not reading the PAEHR was perceived lack of necessity (63%, *n* = 394) and perceived adequacy of information conveyed by the healthcare provider (38%, *n* = 236) (Figure 2). A seventh (14%) of the participants had written other reasons in free text. The most common of them was ‘no particular reason’ or just that they have not had the chance.

## Discussion

To our knowledge, this is the first study to investigate current adoption of PAEHR reading among Swedish primary care patients. Factors related to reading and not reading were also investigated. In our study, most participants reported they were aware they could read the PAEHR, and most reported reading it. Old age, a negative opinion of PAEHR, low information literacy and being single reduced the odds of reported reading; in contrast, access to a smartphone increased use. Most readers reported reading their PAEHR mainly to get an overview of their health and care and/or to follow up on a visit. Most non-readers reported they did not feel a need to read or were satisfied with the information received from their providers.

### Patient-accessible EHR reading

Although awareness was high in the Swedish population compared to other populations,^13^ 14% of the study participants were unaware of the possibility of accessing their EHRs online seven years post-implementation. Further exploration of factors that may influence awareness of ORA in Swedish population is warranted as it may give insights into where interventions focusing on marketing the service could be most useful. Similarly, while the majority of those who were aware had read their records online, 23% had not. The link between awareness and use was also reported in an American study, where 10% of the non-readers of PAEHR had stated lack of awareness as the main reason for not reading.^10^ The level of unawareness might be owed to inadequate communication or marketing efforts; however, the reasons remain unexplored. Healthcare professionals buy-in and promotion could be another important factor influencing awareness and reading. According to another study,^10^ those encouraged to read by their healthcare provider did so more than those not encouraged. In Sweden, as in many other countries, some HCPs reported concerns about the PAEHR implementation including negative effects on the work environment.^28–30^ Addressing these concerns might increase incentives to encourage patients to read, which in turn could increase awareness and reading. In contrast, the relatively high awareness and reading rates reported in our study could be attributed to the COVID-19 pandemic as the national patient portal was used for providing COVID-19 related information, booking of tests, vaccination and receiving lab results.

### Socio-demographic and psychographic factors associated with reading and common reasons for reading and not reading

Age was associated with PAEHR reading in our study. According to other investigations using UTAUT,^19^ while age did not influence PAEHR use in inpatient care, in general practice, it significantly moderated performance- and effort expectancy's influence on intention to use and facilitating conditions’ influence on use.^22,31^ Age could also be associated with digital competency. In a Finnish study, age (60+) was associated with a decline in the use of digital healthcare services. However, high levels of digital competence mitigated this decline up to the age of 80 years.^32^ Another explanation for age being a factor of importance could be traditional views of patient–provider relationships. In an interview study with older Swedes about their views on eHealth, one of the emerging themes was the lack of perceived benefits of eHealth services.^33^

Positive opinions about PAEHR were also associated with online record reading in our study. These opinions could be related to perceived usefulness which has previously been associated with PAEHR use.^16^ This is further emphasized as 63% of the non-readers in our study reported they had no need to read the PAEHR and 38% that they had received enough information from their provider as a reason for not reading. Opinions about PAEHRs could be related to an understanding resulting from benefits of reading. A strategy to increase adoption might be communication about how other patients use the information in their records.

Marital status was another factor associated with PAEHR reading. While no study has investigated the influence of marital status’, being single has previously been identified with non-use or decreased use of patient portals in chronic care.^34^ Marital status could be related to available support to use digital systems such as PAEHR: in one study investigating non-use, those in need of support reported not wanting to seek help and worried about the risks of exposing their records to their spouse.^23^ Therefore, having a partner could be a facilitating condition according to UTAUT. This could be particularly important for people with lower digital literacy or those who struggle with the platform's language. A possible solution to this barrier could be for HCPs to support those who lack this informal support. Marital status may also be related to UTAUT's concept of social influence, where ‘important others’, such as family and HCPs, can impact a person's willingness to use technology.

Additionally, information literacy was found to predict PAEHR reading. Previous studies have associated education, digital- and health literacy to use of PAEHRs.^13^ The broader concept of information literacy reflects aspects included in digital health literacy and likely has a similar impact on PAEHR reading as indicated by our results. In our sample, 5% of the non-readers reported feeling they might not understand the information in their EHR as a reason for their non-use. This concern could be related to effort expectancy which according to UTAUT influences intention and use of technology. The concern for the lack of understanding about their information has previously been raised by non-adopters^23^ and difficulties understanding have been reported.^35^ Support with interpreting the clinical content of PAEHRs could be a potential solution that encourages patient use. In addition, information literacy could be related to a digital literacy and disability prohibiting uptake among some patients. In a Swedish study on disability and the digital divide, people with language, communication, calculation, intellectual and visual impairments reported difficulties using eHealth services such as secure authorization tools and 1177.se patient portal which are gateways to PAEHR use.^36^

We also identified, access to smartphone use as correlated with PAEHR reading. Lack of access to technology has been raised as a barrier to the use of patient portals among older adults.^37^ With an ageing Swedish population, 25% of people aged 65+ report lack of ICT.^38^ In addition, smartphone access could be related to income which has previously been related to PAEHR use.^16^ For some people, the lack of accessible smartphones could be related to the opposition to the digitalization of society in general and the digitalization of healthcare specifically, which has been raised by some non-users.^23^ Further, smartphones are commonly used to access the Swedish patient portal^12^ potentially explaining why access to one increased the odds of reported reading.

Overall, the main reasons for patient reading in our study were consistent with those found in a previous Swedish survey of PAEHR users.^11^ As previously mentioned, the most common reason reported by 65% was reading to get an overview, potentially functioning as a reminder about the patient's medical history. The second most common reason reported, by 55%, was reading to follow-up what was said potentially highlighting issues time limitations and communication constraints in primary care.

When reasons for not reading were investigated, the main reason was not feeling the need to read. These results differ from those from an American study where the main reason was forgetting or being unaware of their ability to read and where a mere 7% thought reading would not be useful.^10^ These differences could be related to differences in the methodology used in each study, the service and the healthcare systems and should be explored further. Further, according to our findings, while some might not feel they need to read, a potentially major barrier to use, reported by 13% of the non-readers, was difficulties using the national patient portal. Difficulties finding the records on the platform were the fifth in the list of reported reason for non-use in the previously mentioned American study.^10^ Difficulties accessing and navigating the Swedish patient portal have also previously been reported including issues using necessary e-identification software to access PAEHR.^39^ In addition, an English study investigating factors influencing the use of patient portals found that the intention to use and facilitating conditions influenced actual use.^31^ Older age, internet access, income and education-level meditated the association between facilitating conditions and use of the patient portal. Moreover, the non-use of patient portals has also previously been related to older age.^40^ Furthermore, the language of the portal and the health record has been shown to impact use.^41^ Since the Swedish PAEHR requires proficiency in Swedish to access and read it, non-Swedish speakers are automatically excluded. Portal usability issues could thus be related to performance expectancy which according to UTAUT influences intention to use. Therefore, improving the usability of patent portals is central to reducing barriers to PAEHR use.

The main takeaways of our investigation are that while a 100% adoption of PAEHRs might be unattainable, there is a digital divide related to PAEHR reading and barriers potentially preventing some people from benefiting from reading their online EHRs which should be addressed.

### Strengths and limitations

Primary care plays a crucial role in the Swedish healthcare system as the first point of contact for patients seeking medical attention. In this study, the accessibility and direct patient engagement of primary care settings facilitated the recruitment of participants during routine visits, ensuring a diverse and representative sample of the general population. Another strength of this study is that it was conducted seven years after implementation of PAEHR, investigating long-term adoption as opposed to the initial adoption of the service. However, as the study has a cross-sectional design we may have data with season-related bias. Participants with certain season-related conditions might thus be over- or underrepresented, however to compensate for COVID-19-related biases, patients seeking care for COVID-19 were not offered to participate in the study.

The survey was not fully pilot-tested which might have influenced the comprehension, yet the extensive cognitive interviews performed before launching the survey only resulted in minor changes indicating that survey was easy to understand. There may also be a recall bias regarding awareness and reading, non-readers might have read and forgotten and the other way around. Moreover, there may be an information bias as this study relied on self-reported information. Additionally, the survey did not include a question about chronic illness or language proficiency, which is a limitation since previous research has highlighted these as important factors influencing usage.^11,41^ However, this study did survey the participants’ general health but found no association with reading PAEHR.

The sample might be partially representative of the adult Swedish population. The focus on the Stockholm region excluded the perspectives of patients in rural areas where, for example, access to healthcare may be lower than in the capital. The online care applications and healthcare centres that we used for this study encompassed the main Swedish online care providers and serve communities with diverse socio-economic status. Telephone consultations were excluded from the study which is a limitation. More effort could also have been made to include people excluded due to language or a disability, for example, by having an option to participate through an interview. The reception staffs’ and online care providers’ way of recruiting the participants might also have influenced the sample.

Finally, the present study has not yet explored the frequency of PAEHR reading. Some people could have read only once, while others read to prepare for every healthcare visit. We also recognize that some of the statements in the survey might increase risk of acquiescence bias as participants are more inclined to agree with positively phrased statements. The study also lacked a true response rate, and we could not calculate the response rate or analyse the difference between responders and non-responders as this data was not collected. In this sample, most participants were females, which could reflect a gender-related bias in our sample.

### Implication and future work

The implications of our study in Sweden underscore the need for incremental and considered integration of PAEHR within the global digital health landscape. While the adoption of PAEHR in Sweden is encouraging, with a high level of patient use, the persistent digital divide due to age, information literacy and device accessibility signals areas for targeted improvement. These findings suggest that it is critical to develop educational programs and support networks that cater to the digitally disenfranchised, particularly among older populations.

Additionally, our study indicates that global health systems may benefit from leveraging mobile technology, a significant facilitator of PAEHR use in Sweden according to our study. This approach could enhance patient engagement by offering more usable and accessible digital health solutions. The Swedish experience provides valuable insights into promoting PAEHR adoption; these can guide global health systems towards more equitable and patient-centered care.

Evidence indicates patient technical training and assistance programmes are currently best in increasing patient portal use in vulnerable groups.^42^ These strategies could thus be employed to enhance utilization of PAEHR. Moreover, HCP encouragement has been indicated to increase PAEHR frequency of use among Swedish adolescents.^43^ Training and informational activities for HCPs are thus also recommended. These could include training HCPs to inform patients about the PAEHR and encourage them to read. It is essential that these training and informational activities are tailored to the specific context of each setting. Further, as portal usability was a barrier to use, improving the portals usability is also recommended. Notably, our study did not investigate previous or current activities aimed at increasing awareness and use of PAEHR, nor did it assess their potential effects.

Our study's implications resonate with findings from the Arabian Gulf, where Tabche et al. (2023) noted the balance EHRs strike in doctor–patient dynamics.^44^ As we consider PAEHR global integration, ensuring these systems support, rather than complicate, these vital relationships are essential. Tailoring EHRs to fit within different cultural contexts while providing appropriate training for HCPs will be key to preserving the integrity of patient care.

Further investigation using UTAUT could illuminate factors influencing use. For example, future work could investigate the infrastructure supporting PAEHR use and the factors influence patients’ ability to access and use PAEHR. Additionally, the influence of gender requires further investigation; notably this is an influential factor according to UTAUT but was not associated with PAEHR reading according to our study. A deeper exploration of PAEHR use in different socio-economic groups could also be the subject of future work.

Ultimately, investing in increased PAEHR use globally can be rewarding for patients, healthcare systems and society, as PAEHR has many benefits^9^ and has the potential to increase health literacy and improve health.^45^

## Conclusions

Seven years after the implementation of PAEHR, most primary care patients in our study were aware that they could read their EHR online but about a sixth were not. Of those aware, the majority had read their EHR online but about a fourth had never read it. While some did not feel they needed to read, there are reports of potential barriers preventing some patient groups from benefiting from reading their online EHRs. Efforts to increase the use of PAEHRs should focus on older adults, individuals who rely more on informal support, people who might be left out because they have limited digital skills, and on making patient portals easier to use.

## Appendix A. The variables

**Table:**

Variable	Questions	Options	Analysed categories	Source
Awareness	Do you know that you as a patient or guardian have access to the medical record via 1177?	Yes No	Yes No	Formulated by the authors of this study
PAEHR reading	Have you read the medical record (care staff's notes, test results, etc.) via 1177?	Yes No	Yes (Has read) No (Has never read)	Formulated by the authors of this study
Gender	Gender?	Female Male Other	Female Male	A British GP patient survey^24^
Age	What year were you born?		2020-birth year	The National Swedish Public Health Survey^26^
Education	What is your highest completed education?	Primary education or similar High school 2 years High school 3–4 years adult school University or college <2 years University or college 3 years or longer Don’t know/don’t want to answer	Primary education or similar High school 2 years & high school 3–4 years & adult school University or college <2 years & university or college 3 years or longer	‘–’
Marital status	What is your current marital status?	Married or registered partner Stable partner Single Other	Married or registered partner, stable partner Single	‘–’
Health	How do you assess your general health?	Very gGood Very Ggood Neither good or bad Bad Very bad	Good or very good Neither good or bad Bad or very bad	‘–’
Accessible devices	Which of the following do you have access to?	Phone without apps Smartphone Computer Tablet Non	Smartphone Just phone without apps, computer, tablet or non	The annual Swedish survey ‘The Swedes and the Internet’^25^
Internet use	How often do you use internet with the following devices? (mobile phone, computer, tablet)	Daily Every week Every month Less frequently Never	Daily Every week or Every month, Sometimes or Never	‘–’
Information Literacy i.e., the ability to retrieve health information, select, understand and judge the reliability of information.	A. I can retrieve information about my health from several different sources of information such as newspapers, internet, books, health care, family and friends, etc. B. I can select exactly the information I need from a variety of information sources C. I can understand the information and share it with others D. I can judge if the information is reliable	Strongly agree Agree Partially agree Disagree Strongly disagree	Strongly agree/agree (High) Partially agree, disagree/strongly disagree (Low)	Adapted from a national patient survey on PAEHR use^11^
It is good for me to read	It is good for me to have access to the medical record on 1177	Strongly agree Agree Partially agree Disagree Strongly disagree	Strongly agree/agree Partially agree, disagree/strongly disagree	‘–’
